# On the Move in Cattle Country: Tracking Nomadic Pastoralists in Southwest Ethiopia

**DOI:** 10.4269/ajtmh.18-0086

**Published:** 2018-07

**Authors:** Hannah Wild

**Affiliations:** Stanford University School of Medicine, Stanford, California

In the summer of 2017, our research team headed to Ethiopia’s remote lower Omo Valley, piloting methods to assess the health of nomadic pastoralists. The sun was still low at the time of day the Nyangatom people call *esimokunyuk*, meaning literally: “the ground squirrel is sweating.” Although only seven, perhaps 8 o’clock in the morning, already the sun beat the hard-packed earth and goats huddled together vying for the scant shade of thorn acacia trees as we searched for one of the Nyangatom’s cattle encampments to conduct interviews on maternal and child health.

Sara Randall, a demographer at University College London, calls mobile pastoralists “invisible” in population data such as censuses, and a World Health Organization–United Nations Children’s Fund–Ethiopian Federal Ministry of Health report showed that systematic data on the health status of pastoralists were “practically nonexistent.” This lack of baseline data seriously hinders efforts to provision these groups with health services. In the push for universal health coverage, pastoralists are a missing denominator.

The mood of the field team was low that day. As with every morning, we had departed before dawn. We needed to reach the camps before the women set out to chase birds away from their precious fields of sorghum seed, cultivatable only a couple of times a year when the dry riverbed flows with rain.

Teaming up with the Stanford Geospatial Center, we used remote sensing and geospatial analysis to locate the current position of the Nyangatom’s mobile cattle camps over large swaths of remote and inaccessible terrain. Relying on a list of global positioning system coordinates, cranky navigation equipment, and the advice of sympathetic young shepherds, tracking down herders in these nomadic settlements was an entirely different enterprise than identifying them from satellite imagery projected onto the walls of Stanford’s gorgeous Rumsey Map Center.

If the women departed for their gardens, we would have to follow them out to the fields, carrying our clunky equipment from woman to woman across acres. Few things eroded morale faster than traipsing through fields lugging hefty wooden height boards and SECA scales. The sweltering sun compounded these less-than-ideal conditions. We craned our necks upward, shouting to respondents as they stood watch over their fields atop crudely constructed wooden platforms or termite mounds. The women issued deafening shrieks to shoo birds away from their crop with a shrill “*Hrrrrrrr!*” that punctuated the interview at irregular intervals, startling me half to death every time.

Field conditions had already taken a heavy toll on our local team: nearly every morning I woke to a text message bearing tidings of a new personnel crisis in an impassioned, frequently poetic resignation letter: “*…you need to be remembering that we too are humans with flesh…*”

Our staff from the relatively comfortable lifestyles of highland towns were burning out faster than birch kindling dealing with the Nyangatom’s high degree of mobility, the remote terrain they inhabit, fluid domestic arrangements, and cultural barriers. However, the staff’s perceived sense of hardship appeared paltry compared with the daily grind the Nyangatom face. They and other nomadic pastoralists are among sub-Saharan Africa’s poorest, hardest-to-reach, and most marginalized populations, often subsisting in the face of drought and conflict. They are also among the least likely to be represented in the data used to plan health interventions.

As the clock ticked, we traced and retraced our route, trying to find a pass through the impenetrable wall of scrub brush blocking us from our target. The undergrowth was full of thorns substantial enough to puncture the tires of a 4 × 4 vehicle, and each new excursion off of our tracks came with risk. Before long, we saw clouds of dust that signaled livestock on the move—not a good sign! If the migrating livestock belonged to the inhabitants of our selected settlement, we might have missed their departure by merely a couple of hours.

We soon came upon the tail of the migration party and began weaving our way through herds of cattle, coaxing testy long-horned bulls out of our path by pounding on the already dented sides of the decrepit Land Rover. Disheartenment set in: the presence of livestock on the trail suggested the camps we were looking for had packed up to migrate in just the few days since our initial scouting mission.

Spotting a herdsman among the cattle, I greeted him and shouted out the window—“And your women? Can they wait to talk with us? We have tea!” I sounded like a desperate salesman.

“They are tying up their saddlebags, little girl!” he shouted in reply.

And so they were, gathered at the perimeter of their village in the distance. Ahead, young men drove teams of donkeys laden with sacks of grain, grinding stones, and toddlers in saddles woven from strips of buffalo hide and saplings. All that the Nyangatom possessed they now carried on these pack animals, or on their own heads and backs.

Now they would travel for days, moving to follow grazing lands and water sources before settling and rebuilding their huts anew. On these journeys, they often walked long into the night. There was little talking. The monotonous clanging of cowbells would be broken only when chaos and bleating erupted as hyenas wove in and out of their ranks in the dark, picking off goats before the men could deter them. When the party stopped for several hours of sleep, women quickly felled brush to pull around themselves, constructing a crude thorn perimeter to keep livestock in and, hopefully, predators out.

On a previous trip, sleeping lightly in one of these makeshift kraals, I asked one of the men: “Can hyenas breach the fence?”

“No,” he answered, correcting me: “*Leopards* climb up over the fence. Hyenas *akipediped*—pierce through it at the base.”

Back in the bush, picking our way through interminable ribbons of cattle, we finally arrived at the selected settlement. The camp was bustling with activity as the remaining families prepared their animals and property to migrate, but novelty worked on our side: curiosity and the promise of tea made a brief delay of their departure a straightforward sell.

Unburdened by any sense of what needed to take place, the enthusiastic patriarch began mobilizing the women and giving orders on our behalf. “You!” he barked at a stupefied child, “Stand on that scale so she can see how many years old you are!”

We sighed in relief, as on other occasions it had required significant diplomacy and improvization to gain admission to villages under the command of less supportive gatekeepers. Once, we were allowed in only after a Hail Mary attempt to win over an elder using my knowledge of an old Nyangatom song for an antelope known as an *egete*. As this influential headman stubbornly rebuffed our initial greetings, I observed that he was sewing a collar for his favorite bull out of goat hide rubbed down with donkey dung, using an *egete* horn as a hook. In a last-ditch effort to break the ice, I began singing a song for the animal that even he had half-forgotten: “*The full moon has come and still he hasn’t had a drop to drink/Oh, he’s dying of thirst,/Gazelle of the tall horns!*” Delighted, he ushered us in to proceed.

By this point in the summer, our interviews went quickly, as we had ironed out most of the wrinkles with which we were quickly confronted at the outset of our work. The women had left little doubt as to what questions they found nonsensical, responding sharply and with great disdain: “Isn’t it you, the people of paper, who know things like that?”

As we prepared to leave, women emerged from their huts offering calabashes full of murky tea full of cows’ eyelashes and dead flies. At the moment, it seemed a great delicacy—one last drink before the move.

Pulling out of the village, the Land Rover’s engine turned over a few times before sputtering to life like an animal out of hibernation. We crested a dip in the sand when it sputtered again, cut out, and unmistakably died.

A pall fell over the crew.

Out of nowhere, women appeared wearing goat skin skirts fringed with ostrich egg shells and ropes of beads made from jujube tree seeds around their necks. They gathered around the vehicle, suggesting they pour the water they had just fetched into the fuel tank to make it run. It was going to be a long, long day.

The team dreaded few things as much as a breakdown out by our most remote settlement. Ahead of us lay a sweltering, many kilometers walk through the bush, following cattle tracks to the nearest point at which someone could bring us the necessary part, or extract us in the back of a crammed Isuzu rumbling across the desert toward the Kenyan border.

Such logistical obstacles are partly what have delayed the systematic implementation of health surveillance and services for mobile pastoralists. A 2012 pilot by researchers at the World Bank of geospatial sampling methods among similar nomadic pastoralists reported “unexpected difficulties,” including “one incident where a team had to walk 15 km to reach the selected site…active volcanoes…minor assaults on drivers and fieldworkers, and the (brief) kidnapping of the survey coordinator.”

Although it is true that fieldwork with nomadic pastoralists brings with it a unique set of challenges, technological tools exist to help cope with these challenges. As climactic stressors, conflict, and humanitarian crises further pressure these groups who already subsist in some of the harshest environments on the planet, a better solution is urgently required.

With mounting concerns about biosecurity and emerging infectious diseases—more than 70% of which are zoonotic in origin—the development of health systems for nomads should be high priority for us all. Improving the quality of data on nomadic populations is a crucial step toward designing health systems for mobile groups, and the conventional census-based sampling frames used for large-scale data collection exercises should be supplemented with alternative sampling frames in pastoralist regions.

The global health community must seek creative solutions to make sure that when we consider “universal health coverage,” the denominator includes everyone.

